# Acute Biomechanical Effects of Empagliflozin on Living Isolated Human Heart Failure Myocardium

**DOI:** 10.1007/s10557-023-07434-3

**Published:** 2023-02-13

**Authors:** Jorik H. Amesz, Sanne J. J. Langmuur, Nina Epskamp, Ad J. J. C. Bogers, Natasja M. S. de Groot, Olivier C. Manintveld, Yannick J. H. J. Taverne

**Affiliations:** 1 Translational Cardiothoracic Surgery Research Lab, Department of Cardiothoracic Surgery, Erasmus University Medical Center, Dr. Molewaterplein 40, 3015GD Rotterdam, the Netherlands; 2 Lowlands Institute for Bioelectric Medicine, Department of Cardiology, Erasmus University Medical Center, Rotterdam, the Netherlands; 3 Department of Cardiology, Erasmus University Medical Center, Rotterdam, the Netherlands; 4 Erasmus MC Transplant Institute, Erasmus University Medical Center, Rotterdam, the Netherlands

**Keywords:** SGLT2 inhibitors, Heart failure, Myocardial slices, Translational research

## Abstract

**Purpose:**

Multiple randomized controlled trials have presented SGLT2 inhibitors (SGLT2i) as novel pharmacological therapy for patients with heart failure, resulting in reductions in hospitalization for heart failure and mortality. Given the absence of SGLT2 receptors in the heart, mechanisms of direct cardioprotective effects of SGLT2i are complex and remain to be investigated. In this study, we evaluated the direct biomechanical effects of SGLT2i empagliflozin on isolated myocardium from end-stage heart failure patients.

**Methods:**

Ventricular tissue biopsies obtained from 7 patients undergoing heart transplantation or ventricular assist device implantation surgery were cut into 27 living myocardial slices (LMS) and mounted in custom-made cultivation chambers with mechanical preload and electrical stimulation, resulting in cardiac contractions. These 300 µm thick LMS were subjected to 10 µM empagliflozin and with continuous recording of biomechanical parameters.

**Results:**

Empagliflozin did not affect the maximum contraction force of the slices, however, increased total contraction duration by 13% (*p* = 0.002) which was determined by prolonged time to peak and time to relaxation (*p* = 0.009 and *p* = 0.003, respectively).

**Conclusion:**

The addition of empagliflozin to LMS from end-stage heart failure patients cultured in a biomimetic system improves contraction and relaxation kinetics by increasing total contraction duration without diminishing maximum force production. Therefore, we present convincing evidence that SGLT2i can directly act on the myocardium in absence of systemic influences from other organ systems.

**Supplementary Information:**

The online version contains supplementary material available at 10.1007/s10557-023-07434-3.

## Introduction

Sodium-glucose cotransporter-2 inhibitors (SGLT2i) have been presented as novel pharmacological therapy for patients with heart failure (HF), showing reductions in hospitalization for HF and mortality in multiple randomized controlled trials [1–5]. These “wonder drugs” were originally designed as anti-diabetic agents inhibiting renal glucose reabsorption, but also showed beneficial cardioprotective and cardiac contractile effects [6]. However, expression of SGLT2 receptors in the heart is absent, and SGLT2i are highly selective, with empagliflozin being the most selective and sotagliflozin showing some inhibitory effect on SGLT1 receptors in the myocardium [7–9]. Yet, mechanisms of direct cardioprotective actions are unclear and remain to be investigated [10].

Several theories have been proposed to explain the direct cardiac effects of SGLT2i therapy [10], including inhibition of myocardial Na^+^/H^+^ exchange [11–14], reduction of calmodulin-dependent kinase II [15, 16], increased phosphorylation levels of myofilament regulatory proteins [17], reduction of cardiac inflammation [18–20], resulting in prevention of adverse cardiac remodeling [21, 22], and reduction of fibrosis [23, 24]. However, current evidence is conflicting [25, 26], and most studies have been performed on two-dimensional cardiomyocyte cultures or animal models, which complicates extrapolation to the intact human heart.

Living myocardial slices (LMS) are isolated sections of patient-specific myocardium [27] that are directly produced from human ventricular material with intact three-dimensional architecture, extracellular matrix, and cell–cell interactions [28]. In the current study, we aimed to evaluate whether SGLT2i empagliflozin has a direct effect on the myocardial contractility of LMS from end-stage HF patients.

## Methods

### Tissue Acquisition

Cardiac tissue was obtained from patients with end-stage HF undergoing surgery for cardiac transplantation or ventricular assist device implantation. All patients provided informed consent for use of their explanted tissue for scientific research, as approved by the Medical Ethical Committee of the Erasmus Medical Center (MEC 2020–0988). Ventricular specimens were immediately submerged in 4 °C Tyrode slicing buffer (NaCl 136 mM, KCl 5.4 mM, MgCl_2_*6H_2_O 1 mM, NaH_2_PO_4_*H_2_O 0.33 mM, glucose 10 mM, CaCl_2_*2H_2_O 0.9 mM, 2,3-butanedione monoxime 30 mM, HEPES 5 mM, pH 7.4) and transported on ice to the laboratory.

### Slice Production

The technique to produce LMS has been previously described in detail by Fischer et al. [28, 29]. In short, epicardial fat and excessive endocardial trabeculae were removed, and tissue specimens were trimmed to a cross-sectional area of approximately 1 × 1 cm^2^. This tissue block was submerged with the epicardial side down in 37 °C 4% low-melting agarose (Agarose II, VWR Chemicals LLC, Solon, OH, USA) and cooled until the gel solidified. The embedded tissue was placed in a 4 °C Tyrode buffer-filled bath of a high-precision cutting vibratome (VT1200S, Leica BioSystems, Nussloch, Germany) which produced slices with a thickness of 300 µm (settings: vibration amplitude 1.3 mm, blade advance speed 0.07 mm/s). The surrounding agarose was then removed from the slices, and miniature plastic triangles were glued to both ends of the slices, with longitudinally aligned fiber orientation in between. Slices were mounted in custom-made biomimetic cultivation chambers (BMCCs) (InVitroSys GmbH, Munich, Germany) [28], and a diastolic preload of ≈1 mN was applied by providing mechanical stretch to the tissue, corresponding to a sarcomere length of ≈1.8 µm and normal mean diastolic wall stress of 0.66 kN/m^2^ [28, 30]. Electrical field stimulation (output 50 mA, pulse duration 3.0 ms) resulted in an isotonic contraction of the LMS.

### Slice Cultivation

BMCCs were filled with 2.4 mL of 37 °C culture medium (Gibco Medium-199 (Grand Island, NY, USA) supplemented with 5% penicillin–streptomycin, 5% Insulin-Transferrin-Selenium-X and 50 µM 2-Mercaptoethanol), and the medium was refreshed after 1 h and subsequently every 24 h by replacing 1.6 mL in the BMCC. Preload was readjusted to 1 mN at the first two medium exchanges. BMCCs were placed in a standard 37 °C 5% CO_2_ incubator and placed on a rocking plate (30 rpm) for continuous agitation. LMS were electrically stimulated at 0.5 Hz.

### Empagliflozin Addition

Empagliflozin tablets (Jardiance 10 mg, Boehringer Ingelheim, Ingelheim am Rhein, Germany) were dissolved in dimethyl sulfoxide solvent (DMSO) (ChemCruz DMSO Cell Culture reagent, Santa Cruz Biotechnology, Dallas, TX, USA) to a final concentration of 50 mg/mL. This solution was further diluted in the culture medium, and 4 µL were added to the BMCC to reach a final concentration of 10 µM in the BMCC, equivalent to human subjects taking 50 mg empagliflozin [31]. The final concentration of DMSO in the BMCCs was < 0.01%. Electromechanical cultivation conditions (stimulation frequency: 0.5 Hz, diastolic preload: ≈1 mN) were identical before and after the addition of empagliflozin.

### Data Acquisition

**Fig. 1:**
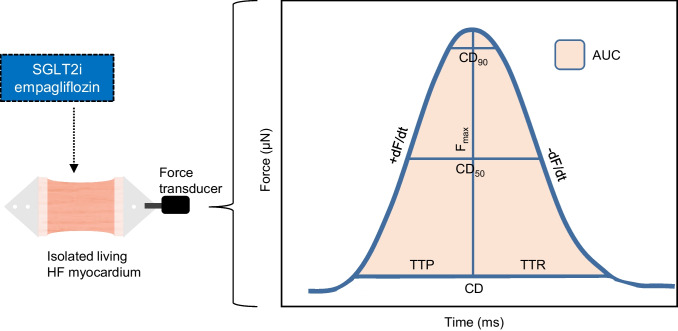
Definitions of contractility parameters which were measured with a magnetic force transducer before and after the addition of empagliflozin. *AUC*, area under the curve; *CD*, total contraction duration; *CD*_*50*_, peak width at 50% of the maximum amplitude; *CD*_*90*_, peak width at 90% of the maximum amplitude; *F*_*max*_, maximum contraction force; *TTP*, time to peak; *TTR*, time to relaxation; *+ dF/dt*, steepest positive slope; *− dF/dt*, steepest negative slope

The contraction force was continuously measured by a magnetic force transducer in the BMCC [28]. Contractility was analyzed before and 10 min after the addition of empagliflozin by averaging 30 s of data. For each contraction, force amplitude (F_max_), peak area (AUC), contraction duration (CD), peak width at 50% of the maximum amplitude (CD_50_), time to peak (TTP), time to relaxation (TTR), steepest positive slope (+ dF/dt), and steepest negative slope (− dF/dt) were extracted from the system recordings with the peak analysis module of LabChart 8 software (ADInstruments). Parameters were defined as shown in Fig. 1. The start and end of the peak were chosen at 10% away from the baseline to compensate for baseline noise.

### Statistics

The median and interquartile range (IQR) were calculated for all contraction parameters. Outliers were excluded from analysis if the contraction force was > 10 mN. For comparison of values before and after the addition of empagliflozin, a Wilcoxon Signed Rank test was executed in SPSS (IBM SPSS Statistics version 28.0.1.0 (142)). *P*-values were considered statistically significant if *P* < 0.05.

## Results

### LMS Characteristics

Ventricular specimens were obtained from 7 end-stage HF patients undergoing cardiac transplantation or left ventricle assist device implantation. In total, 27 beating LMS were produced from these biopsies. Patient and LMS characteristics are presented in Table 1. The underlying disease encompassed 6 non-ischemic cardiomyopathies and 1 ischemic cardiomyopathy. One patient was using 10 mg of dapagliflozin daily, which was stopped 10 days prior to surgery.

**Table 1:** Patient and living myocardial slice (LMS) characteristics

Patient characteristics	*N* = 7
Age, years	46 ± 15
Male, *n* (%)	5 (71%)
Diabetes mellitus, *n* (%)	1 (14%)
Etiology of heart failure	
Ischemic cardiomyopathy, *n* (%)	1 (14%)
Dilated cardiomyopathy, *n* (%)	3 (43%)
Non-compaction cardiomyopathy, *n* (%)	1 (14%)
Congenital heart disease, *n* (%)	1 (14%)
Post-partum cardiomyopathy, *n* (%)	1 (14%)
Surgery	
LVAD implantation, *n* (%)	3 (43%)
Heart transplant, *n* (%)	4 (57%)
Medication	
SGLT2 inhibitor, *n* (%)	1 (14%)
LMS characteristics	*N* = 27
Ventricle	
Left, *n* (%)	16 (59%)
Right, *n* (%)	11 (41%)

### Effect of Empagliflozin on Contractility

**Fig. 2:**
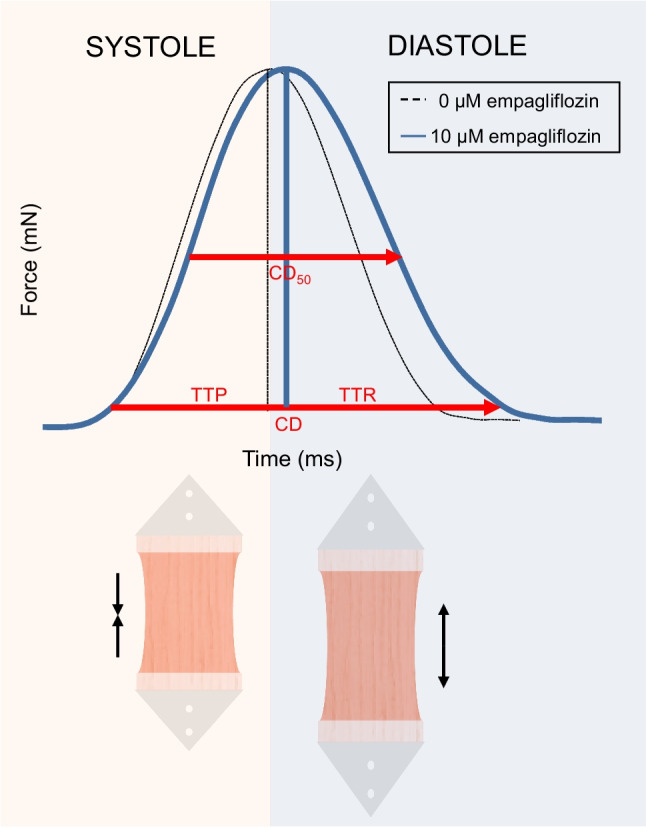
Addition of empagliflozin to living myocardial slices (LMS) from patients with heart failure resulted in significantly prolonged contraction duration (526.7 ms (424.7–668.7) vs. 595.3 (427.7–702.4), *p* = 0.002). Systolic time (TTP = upward phase of the contraction peak when the LMS shortens in length), and diastolic time (TTR = downward phase of the contraction peak when LMS relaxes to original state) both increased significantly, with an increase in CD_50_, however maintaining the same CD_90_ and AUC. *CD*, total contraction duration; *CD*_*50*_, peak width at 50% of the maximum amplitude; *LMS*, living myocardial slices; *TTP*, time to peak; *TTR*, time to relaxation

The addition of empagliflozin did not alter contraction force, however, resulted in a statistically significant increase of 13% in median CD. The longer CD is determined by prolonged TTP (+ 7%) and a slightly larger increased TTR (+ 10%) (*p* = 0.009 and *p* = 0.003, respectively). This effect can be seen in Table 2 and Fig. 2 and was consistently observed in all patients. Exemplary contractility traces can be found in the Supplementary Material. Seven LMS obtained from different patients had no reaction to empagliflozin despite normal contractile biomechanics as compared to the other LMS. Empagliflozin acted on both left (CD: 588.0 ms (415.2–716.9) vs. 618.3 ms (428.5–766.3), *p* = 0.049) and right ventricular LMS (CD: 521.3 ms (436.0–565.3) vs. 595.3 ms (439.4–655.4), *p* = 0.016). CD appeared longer in the left ventricular LMS in comparison with the right ventricular LMS before and after the addition of empagliflozin. Increases in CD were observed at 50% of the F_max_, but not at 90% of the F_max_. The effects of empagliflozin showed no acute directional changes in the steepness of contraction and relaxation over time (dF/dt) and the AUC stayed the same. Presented biomechanical profile alterations were consistent throughout all underlying diseases and irrespective of the presence of diabetes.

**Table 2:** Effect of 10 µM empagliflozin addition on contractility of isolated HF LMS (*n* = 27)

Parameter	0 uM empagliflozin (*n* = 27)	10 uM empagliflozin (*n* = 27)	*P* value
F_max_ (µN)	727.7 (445.0–1641)	726.9 (431.3–1661.5)	0.124
CD (ms)	526.7 (424.7–668.7)	595.3 (427.7–702.4)	0.002
CD_50_ (ms)	268.7 (235–291.7)	288 (235–331.7)	0.003
CD_90_ (ms)	92.3 (72.2–111.7)	93.3 (80.8–110.0)	0.346
TTP (ms)	208.7 (171.4–225.3)	224.0 (176–246.3)	0.009
TTR (ms)	328.0 (250.0–454.4)	360.6 (249.4–483.4)	0.003
AUC (µN⋅s)	199.4 (112.4–530.7)	258.0 (122.9–634.0)	0.113
+ dF/dt (µN/ms)	5820 (3450–13,125)	4782 (3170–12,810)	0.701
-dF/dt (µN/ms)	− 4235 (− 10161– − 2525)	− 3715 (− 10177– − 2649)	0.962

Data expressed as median (interquartile range). CD, CD_50_, TTR, and TTP significantly increased after the addition of empagliflozin. *AUC*, area under the curve; *CD*, total contraction duration; *CD*_*50*_, peak width at 50% of the maximum amplitude; *CD*_*90*_, peak width at 90% of the maximum amplitude; *F*_*max*_, maximum contraction force; *TTP*, time to peak; *TTR*, time to relaxation; + *dF/dt*, steepest positive slope; − *dF/dt*, steepest negative slope

## Discussion

To our knowledge, this is the first study to evaluate and confirm the direct effects of SGLT2i on myocardial contractility using LMS in a biomimetic system. The addition of empagliflozin to LMS from end-stage HF patients increased total CD, resulting from an increase in the duration of both the systolic and diastolic phase of the contraction, without diminishing maximum force production or altering force displacement.

### (Direct) Effects of SGLT2i on Cardiac Contractility

SGLT2i have been developed to treat hyperglycemia in patients with type II diabetes mellitus, which act by inhibiting glucose reabsorption in the proximal tubule of the kidney [10]. Ensuing studies have highlighted important effects outside the kidney, suggesting potential new pharmacological targets for HF patients [1–6]. Of note, it is important to make a clear distinction between specific myocardial effects and systemic effects that alter cardiac function when investigating the mechanistic properties of SGLT2i [17]. In-vivo studies are therefore, despite their great value in multidirectional analyses, less suited to study the direct effects of SGLT2i on myocardial contraction. LMS in an advanced setup, like this biomimetic system, allow for an isolated and controlled setting [27] to assess the biomechanical effects of SGLT2i. In this study, culture conditions were equal before and after drug administration and each LMS served as its own control, which is why the effects of empagliflozin addition are likely to be a direct effect of empagliflozin on the myocardium.

In our study, both contraction and relaxation duration were prolonged while maintaining maximum force generation per beat, consequently seen in all patients regardless of underlying etiology of heart failure. In order to comprehend these results, a number of biomechanical parameters need to be addressed. LMS in culture show isotonic contractions where the same tension is maintained while the muscle slice shortens. As presented in Fig. 2, there is a shift and alteration of the form of the curve due to the increase in time needed to contract (TTP) and an even longer time needed to relax (TTR), yet, without altering the F_max_ or dF/dt nor a change in the AUC. In other words, the effect seen can best be described as a more gradual build-up and off of force over time, and thus more efficient handling of contraction and relaxation kinetics.

Furthermore, the observed prolongation of CD appeared irrespective of the type of underlying cardiomyopathy, although the sample size was low and this was not statistically tested. The longer median CD of left ventricular LMS compared to right ventricular LMS is possibly explained by micro-architectural and physiological differences between the left and right ventricle originating from dissimilarities in their development, anatomy, and function [32]. The fact that 7 LMS from different patients did not react to empagliflozin administration whilst exhibiting a normal contractile profile might be based on inter-layer variability of the myocardium with LMS production.

### Biomechanical Profile of Empagliflozin

The observed shift in diastolic function corroborates with findings from previous studies [17, 21]. Pabel et al. showed a ~24.2% decrease in diastolic tension of human and murine twitching ventricular trabeculae after empagliflozin administration, while diastolic tension remained unaffected in controls treated with DMSO [17]. The systolic force did not change in their study, being similar to our results. However, in our study, the constant maximum contractile force was accompanied by a 7% increase in TTP. Yet, Azam et al. also assessed the direct effect of empagliflozin on cardiac contractility of Langendorff-perfused rabbit hearts subjected to global ischemia–reperfusion and showed improved left ventricular developed pressure without altering left ventricular end-diastolic pressure [33]. The differences between their study and our study might be explained by the different nature of both studies, as they worked with a rabbit model of ischemia–reperfusion and we used human tissue from patients with end-stage HF. Pabel et al. showed that the observed contractile effects can be attributed to a reduction in the myofilament stiffness of cardiomyocytes due to increased phosphorylation of myofilament regulatory proteins [17]. These results were confirmed by in-vivo echocardiographic measurements in rats, showing a shortened isovolumetric relaxation time and increased E/A diastolic filling velocities ratio [17]. Similar results with regard to diastolic function were obtained by animal studies from other groups [21, 34, 35] and in-human magnetic resonance imaging trials [22]. Our study, together with the study from Pabel et al. [17], shows that these effects are independent of systemic effects that may indirectly influence cardiac contractility, since the study was performed on isolated myocardium.

Two important mechanisms are present in the heart to modulate its ability to increase strength and rate of contraction, based on the β-adrenergic pathway and length-dependent activation [36]. Our study consistently showed no effect of empagliflozin on force generation or maximum force attained throughout all patient samples. This implies that secondary empagliflozin effects are not related to the β-adrenergic pathway, as increases in contractile force would then be expected. However, this was not assessed directly in this study. Second, myofilament properties play a central role in cardiac relaxation where protein kinase A (PKA)-mediated myofilament phosphorylation affects length-dependent activation, and the decline of intracellular Ca^2+^ is necessary [36]. Phosphorylation of cardiomyocyte binding protein C has been shown to play a vital role in cardiac diastolic function [37] and recent studies on molecular docking suggested glucose transporters 1 and 4 as potential binding target sites for empagliflozin [38]. This could potentially explain the altered biomechanical kinetics presented in our study where direct binding of empagliflozin with those glucose transporters restores the coupling between glycolysis and oxidative phosphorylation [39]. Nevertheless, such a restored coupling would also show a resultant enhanced Ca^2+^ transient with increased contractility, a feature that was absent in our study. Hence, varying results have been reported on the contractile effects of empagliflozin which could be attributed to the model used. Up-to-date, most studies either used isolated cardiomyocytes thereby lacking three-dimensional microarchitecture and native extracellular matrix, or animal models sometimes lacking extrapolation to the human setting. In addition, studies that used cardiac tissue biopsies [17, 19] mainly depended on isometric twitching of papillary muscles where papillary muscles are known to poorly project ventricular free wall kinetics and isometric testing does not reflect near-physiological human cardiac function [27].

As such, in our study, we opted to use human end-stage heart failure patient-specific tissue for biomimetic electromechanical stimulation with physiological preload that allowed for constant monitoring of the contractile capacity which provided crucial information related to tissue function in real-time. LMS can be kept in culture for several weeks [28], while still presenting a high degree of in-vivo representativeness [27]. Biochemical analysis of the culture medium and slices will hopefully contribute to a better understanding of the molecular mechanisms underlying SGLT2i therapy for patients with HF in future studies.

### Limitations

The current study presents some limitations. Firstly, it assessed the direct effect of empagliflozin on cardiac contractility based on mechanical force measurements, but without molecular analyses. This would be of added value for future studies. Secondly, the effects of DMSO cannot be completely ruled out, since control experiments were not performed. Yet, the final DMSO concentrations were very low. Thirdly, the use of different drugs in individual patients prior to surgery was not taken into account, which could have influenced the contractile response to empagliflozin administration in LMS. However, the setup used the same LMS as controls, and effects were seen in all patients. Furthermore, given the extended handling of LMS (wash-out in Tyrode buffer and culture medium) and the half-time of most HF medications, it is unlikely that receptors would still have been blocked by prior medication. Fourthly, the majority of tissue in this study was obtained from non-diabetic patients (*n* = 6/7), which does not allow for comparison between diabetic and non-diabetic patients. Yet, although only *n* = 1, the increase in CD was also observed in the LMS from the patient with diabetes mellitus. Lastly, no patients with heart failure and preserved ejection fraction (HFpEF) were present in this study and the question rises whether the presented effects could be extrapolated to this population. Yet, the inclusion of patients with HFpEF is very difficult since they do not undergo cardiac surgery very often.

### Clinical Relevance

Data from this study indicates that SGLT2i directly affect cardiac contractility, apart from volume regulation, cardiorenal mechanisms, and metabolic effects, aiding our understanding of the observed beneficial effects in diabetic and non-diabetic patients with HF.

Recently, it has been demonstrated that a shortened systolic ejection time (SET) is independently associated with an increased risk of cardiovascular morbidity and mortality in patients with HF [40, 41]. This supports a potential role for normalizing cardiac time intervals in patients with HF. In the current study, total contraction duration was prolonged after empagliflozin administration, including time-to-peak, which represents the systolic phase of the cardiac contraction. Hence, our data suggest that SGLT2i can directly improve SET intervals, which could correlate to the significant reductions in hospitalizations and mortality as seen in clinical studies of SGLT2i therapy. Yet, more in-vivo imaging studies are needed to confirm whether empagliflozin and other SGLT2i indeed improve cardiac time intervals as suggested by the current study.

### Conclusion

In conclusion, contraction duration of isolated LMS from end-stage HF patients increased after the addition of empagliflozin without diminishing maximum force production. Here, we present convincing evidence that SGLT2i directly act on the myocardium, independent of renal and other systemic influences. Yet, the exact pathway behind this cardioprotective mechanism needs to be investigated in future studies.

### Supplementary Information

Below is the link to the electronic supplementary material.

Supplementary file1 (PDF 152 kb)

## Data Availability

The data underlying this article will be shared on reasonable request to the corresponding author.
