# Supplementary material for: Acute Biomechanical Effects of Empagliflozin on Living Isolated Human Heart Failure Myocardium
Source: Cardiovasc Drugs Ther. 2023 Feb 13;38(4):659–66. doi: 10.1007/s10557-023-07434-3 (PMC11266265; doi:10.1007/s10557-023-07434-3)
Supplement: Supplementary file 1 — Supplementary file1 (PDF 152 kb) [file 10557_2023_7434_MOESM1_ESM.pdf]

Supplementary Material

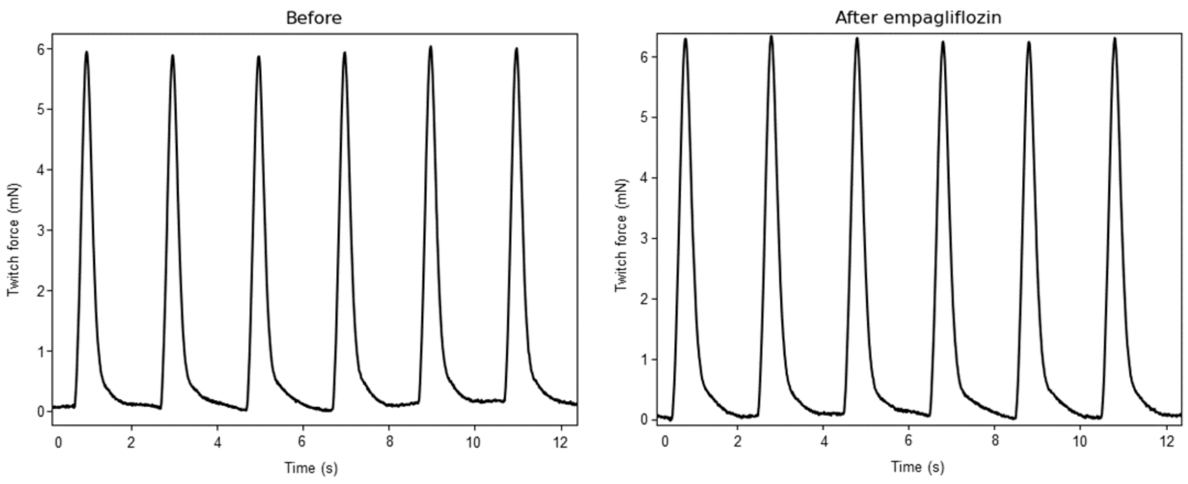

Supplementary Fig. 1. Exemplary contractility tracings before and after addition of 10 µM empagliflozin.
